# Neutrophil‐to‐lymphocyte ratio and platelet‐to‐lymphocyte ratio are associated with disease activity in polymyalgia rheumatica

**DOI:** 10.1002/jcla.23000

**Published:** 2019-08-11

**Authors:** Ju‐Yang Jung, Eunyoung Lee, Chang‐Hee Suh, Hyoun‐Ah Kim

**Affiliations:** ^1^ Department of Rheumatology Ajou University School of Medicine Suwon Korea; ^2^ Department of Biomedical Informatics Ajou University School of Medicine Suwon Korea; ^3^ Office of Biostatistics Ajou Research Institute for Innovative Medicine Suwon Korea

**Keywords:** neutrophil‐to‐lymphocyte ratio, polymyalgia rheumatica, prognosis

## Abstract

**Background:**

The neutrophil‐to‐lymphocyte ratio (NLR), platelet‐to‐lymphocyte ratio (PLR), and monocyte‐to‐lymphocyte ratio (MLR) are indicators of systemic inflammation and are useful as markers in systemic rheumatic diseases. In this study, we compared the NLR, PLR, and MLR among patients with polymyalgia rheumatica (PMR) and rheumatoid arthritis (RA), and explored possible associations with clinical features, disease activity, and prognosis in patients with PMR.

**Methods:**

The study enrolled 94 patients with PMR and 242 patients with RA who were initially diagnosed at the rheumatology clinic of a university‐based tertiary hospital. Symptoms, physical examination, and medical histories were collected with the results of laboratory tests.

**Results:**

Neutrophil‐to‐lymphocyte ratio (4.5 ± 3.3 vs 2.8 ± 1.8), PLR (222.7 ± 115.5 vs 159.7 ± 78.1), and MLR (0.4 ± 0.3 vs 0.3 ± 0.2) were higher in patients with PMR compared with patients with RA (all *P* < .001). NLR, PLR, and MLR were correlated with specific laboratory values, including CRP and albumin, in patients with PMR. After disease activity resolved, NLR (2.95 ± 2.32, *P* < .001), PLR (137.5 ± 82.3, *P* < .001), and MLR (0.26 ± 0.16, *P* < .001) decreased significantly. By comparing patients according to the disease course, swollen joint counts were higher in the chronic course group compared with the remission group (*P* = .03), while the NLR, PLR, and MLR were similar.

**Conclusions:**

Neutrophil‐to‐lymphocyte ratio, platelet‐to‐lymphocyte ratio, and monocyte‐to‐lymphocyte ratio levels were associated with disease activity and specific clinical features, although they could not predict prognosis in patients with PMR.

## 1 INTRODUCTION

Polymyalgia rheumatica (PMR) is a systemic inflammatory disease, typically found in people over 50 years of age, affecting the shoulder and pelvic girdles.^1^, ^2^, ^3^ Typical symptoms include inflammatory pain and stiffness of the involved joints with elevated inflammatory markers, such as the erythrocyte sedimentation rate (ESR), and C‐reactive protein (CRP). There is no specific serological marker or test for PMR, and the diagnosis depends on clinical characteristics. Several classification criteria, including clinical characteristics and imaging tests, have been used.^4^ Ultrasonography is used to identify bursitis or tenosynovitis in affected lesions, and magnetic resonance imaging and positron emission tomography scans with 18F‐fluorodeoxyglucose have been investigated as imaging tools to assess inflammation around large joints in PMR.^5^, ^6^, ^7^ Studies have shown that levels of circulating proinflammatory cytokines, such as interleukin (IL)‐6 and fibrinogen, are increased in active PMR, and their levels represent disease activity.^8^, ^9^, ^10^, ^11^ In addition, levels of several other proinflammatory cytokines, including IL‐1α, IL‐1β, IL‐8, and monocyte chemoattractant protein‐1, are elevated in symptomatic muscles.^12^

Systemic glucocorticoids are the treatment of choice to relieve disease manifestations.^13^ Although PMR is generally considered a benign disorder with no adverse effects on long‐term survival, most patients with PMR require long‐term glucocorticoid therapy, which has several side effects, such as osteoporotic fractures and infection. Moreover, 20%‐55% of patients have relapses in the first year, requiring an increased dose of glucocorticoids.^14^ Immunosuppressant agents including methotrexate (MTX) are added to taper glucocorticoids and prevent relapse.^15^ Clinical characteristics such as female sex, peripheral arthritis, and high ESR or CRP levels are considered risk factors for relapse or prolonged glucocorticoid use.^16^ However, there are few specific markers that predict the disease prognosis.

The neutrophil‐to‐lymphocyte ratio (NLR), platelet‐to‐lymphocyte ratio (PLR), and monocyte‐to‐lymphocyte ratio (MLR) are calculated from the complete blood count (CBC) and are known to represent systemic inflammation.^17^, ^18^ The NLR, PLR, and MLR have been studied in infectious or malignant diseases and have been shown to be useful markers for systemic rheumatic diseases such as rheumatoid arthritis (RA) and systemic lupus erythematosus.^19^, ^20^, ^21^, ^22^ Recent studies have shown that the NLR and PLR are higher in patients with RA than in healthy controls and associated with disease activity in such patients.^18^, ^23^ However, they have never been investigated in patients with PMR.

Therefore, we examined the clinical significance of NLR, PLR, and MLR in Korean patients with PMR. The NLR, PLR, and MLR in patients with PMR were compared with those of patients with RA. In addition, their correlations with disease activity markers, clinical features, and disease course were evaluated.

## 2 METHODS

### 2.1 Subjects

A total of 94 patients with PMR and 242 patients with RA aged 50 years or older who had been initially diagnosed at the rheumatology clinic of university‐based tertiary hospitals were enrolled between January 2008 and June 2018. The diagnosis of PMR was based on Bird's criteria, and a diagnosis of probable PMR was made if more than three of seven criteria were met.^24^ Patients were excluded if they had other rheumatic diseases, including systemic lupus erythematosus, systemic infections, or malignancy. The diagnosis of RA was based on the American College of Rheumatology 1987 revised criteria.^25^ Symptoms, results of physical examination, and medical histories were collected with laboratory test results, such as the CBC, ESR, CRP, rheumatoid factor (RF), anti‐cyclic citrullinated peptides (CCP) antibody, antinuclear antibody (ANA), and liver function tests. All data on disease characteristics were recorded in a standardized form. A visual analogue scale for pain was used as a pain assessment tool. The duration of morning stiffness is the time after waking during which patients feel rigidity of the whole body as a manifestation of PMR. Remission was defined as a state of no disease activity in the articular or systemic manifestations without any drugs or recurrence for at least two consecutive months. Disease flare was defined as deterioration of PMR symptoms that required an increase in glucocorticoid dose. Based on the CBC, which was measured using the ADVIA 2120i Hematology System (Siemens Healthcare Diagnostics), NLR was the proportion of absolute neutrophil‐to‐lymphocyte count, MLR was the proportion of monocyte‐to‐lymphocyte count, and PLR was as the proportion of platelet‐to‐lymphocyte count. The Institutional Review Board of our hospital approved this study, and the need to obtain informed consent was waived because of the retrospective study nature (AJIRB‐MED‐MD8‐18‐265).

### 2.2 Statistical analysis

The chi‐square test was used to compare categorical variables for the clinical characteristics between patients with PMR and RA. The independent *t* test was used to compare the NLR, PLR, and MLR levels according to disease manifestations, and the clinical characteristics of patients with active and inactive PMR. Performing the Shapiro‐Wilk test on all three ratios gave *P*‐values < .0001, which does not indicate a normal distribution. However, each group was sufficiently large to apply the *t* test or chi‐square test. Spearman's correlation was used to assess the correlations between disease activity markers and NLR, PLR, or MLR. The correlation coefficient, r, shows the statistical strength of the relationship between two variables. IBM SPSS 23.0 (SPSS) was used for the statistical analyses.

## 3 RESULTS

### 3.1 Clinical characteristics of patients with PMR and RA

The study enrolled 94 patients with PMR and 242 patients with RA. The mean age at disease onset differed with the diseases and was 64.7 ± 9.7 and 61.4 ± 8.7 years (*P* = .003), respectively (Table 1). No patients with PMR and 129 (76%) patients with RA had anti‐CCP antibody. Symptom duration before presenting to the hospital was shorter in patients with PMR than in patients with RA (5.4 ± 5.8 vs 9.0 ± 10.7 months, *P* = .002). The number of tender and swollen joints was lower, and the visual analogue scale (VAS) score was higher in patients with PMR than in those with RA (all *P* < .001). In terms of the laboratory results, hemoglobin count was lower and the white blood cell (WBC) and platelet counts were higher in patients with PMR than in those with RA (all *P* < .001). Neutrophil count was higher, and lymphocyte count was lower in patients with PMR than in those with RA (all *P* < .001). ESR and CRP levels were higher in patients with PMR than in those with RA (all *P* < .001). Uric acid and albumin levels were lower in patients with PMR than in those with RA (*P* = .034 and *P* < .001).

**Table 1:** Patient clinical characteristics of patients with platelet‐lymphocyte ratio (PLR) and rheumatoid arthritis (RA)

Characteristics	PMR patients (N = 94)	RA patients (N = 242)	*P*‐value
Age at diagnosis, y	64.7 ± 9.7	61.4 ± 8.7	.003
Sex			
Male	21 (23%)	41 (17%)	.23
Female	72 (77%)	201 (83%)	
Smoking, n (%)	12 (13%)	23 (10%)	.36
Alcohol, n (%)	9 (10%)	22 (9%)	.87
Positive RF, n (%)	12 (13%)	214 (88%)	<.001
Positive anti‐CCP antibody, n (%)	0	129 (76%)	<.001
ANA, n (%)	20 (22%)	63 (26%)	.37
Duration of illness to hospital, mo	5.4 ± 5.8	9.0 ± 10.7	.002
Tender joint count	3.9 ± 5.7	9.9 ± 8.2	<.001
Swollen joint count	1.8 ± 4.3	4.4 ± 5.4	<.001
Visual analogue scale for pain	6.5 ± 2.0	5.0 ± 2.1	<.001
WBC, /µL	9365.1 ± 3203.3	7567.5 ± 2295.0	<.001
Hemoglobin, /µL	11.5 ± 1.6	12.6 ± 1.3	<.001
Neutrophil, /µL	6752.3 ± 2880.6	4741.1 ± 2045.3	<.001
Lymphocyte, /µL	1796.7 ± 679.2	2102.0 ± 721.9	<.001
Monocyte, /µL	665.7 ± 528.9	548.3 ± 491.7	.056
RDW, %	14.5 ± 1.9	13.7 ± 1.7	<.001
MPV, fL	7.4 ± 0.9	7.7 ± 1.6	.07
Platelet, ×10^3^/µL	348.1 ± 104.6	286.4 ± 86.3	<.001
ESR, mm/h	73.5 ± 25.6	41.8 ± 27.1	<.001
CRP, mg/dL	6.7 ± 6.2	1.4 ± 2.1	<.001
Uric acid, mg/dL	4.0 ± 1.6	4.4 ± 1.1	.034
Albumin, g/dL	3.9 ± 0.4	4.2 ± 0.4	<.001
ALP, U/L	100.4 ± 68.6	83.0 ± 33.7	.002
AST, U/L	25.5 ± 18.2	26.2 ± 23.1	.81
ALT, U/L	25.1 ± 27.4	24.9 ± 34.9	.96
Bilirubin, mg/dL	0.5 ± 0.3	0.5 ± 0.2	.031

Note

These data were assessed with Pearson's chi‐square test or independent *t* test.

Abbreviations: Ab, Antibody; ALP, Alkaline phosphatase; ALT, Alanine aminotransferase; ANA, Antinuclear antibody; AST, Aspartate transaminase; CCP, Cyclic citrullinated peptide; CRP, C‐reactive protein; ESR, Erythrocyte sedimentation rate; MPV, Mean platelet volume; PMR, Polymyalgia rheumatic; RA, Rheumatoid arthritis; RDW, Red cell distribution width; RF, Rheumatoid factor; WBC, White blood cells.

### 3.2 NLR, PLR, and MLR of patients with PMR and RA

**Figure 1:**
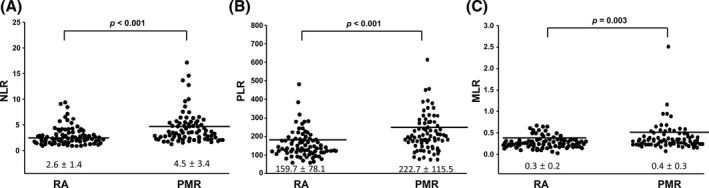
Neutrophil‐to‐lymphocyte ratio (NLR) (A), platelet‐to‐lymphocyte ratio (PLR) (B), and monocyte‐to‐lymphocyte ratio (MLR) (C) in 94 patients with polymyalgia rheumatica (PMR) and 94 patients with rheumatoid arthritis (RA) matched by age and sex. Data were expressed as the mean ± SD. An independent *t* test was used for statistical analysis

Table 2 shows the levels of NLR, PLR, and MLR in patients with PMR and RA. When they were matched by age and gender, NLR was 4.5 ± 3.4 in patients with PMR and 2.6 ± 1.4 in patients with RA (*P* < .001, Figure 1A), PLR was 222.7 ± 115.5 in patients with PMR and 159.7 ± 78.1 in patients with RA (*P* < .001, Figure 1B), and MLR was 0.4 ± 0.3 in patients with PMR and 0.3 ± 0.2 in patients with RA (*P* = .003, Figure 1C). When they were matched by ESR and CRP, NLR, PLR, and MLR did not differ between the patients with PMR and RA.

**Table 2:** Comparison of neutrophil‐lymphocyte ratio, platelet‐lymphocyte ratio, and monocyte‐lymphocyte ratio between patients with polymyalgia rheumatic (PMR) and rheumatoid arthritis (RA)

Characteristics	PMR	RA	*P*‐value
Matched by age and sex			
Number	94	94	
NLR	4.5 ± 3.3	2.8 ± 1.8	<.001
PLR	222.7 ± 115.5	159.7 ± 78.1	<.001
MLR	0.4 ± 0.3	0.3 ± 0.2	.003
RDW, %	14.5 ± 1.9	13.7 ± 1.6	.003
MPV, fL	7.4 ± 0.9	7.6 ± 1.1	.1
Hb, /µL	11.5 ± 1.6	12.6 ± 1.4	<.001
WBC, /µL	9327.1 ± 3207.2	7681.5 ± 2483.6	<.001
Matching with ESR and CRP			
Number	64	64	
NLR	3.6 ± 2.1	3.0 ± 1.9	.13
PLR	206.1 ± 116.2	183.8 ± 75.9	.2
MLR	0.3 ± 0.1	0.4 ± 0.4	.44
RDW, %	14.5 ± 2.0	14.1 ± 2.1	.26
MPV, fL	7.4 ± 1.0	7.3 ± 0.9	.31
Hb, /µL	11.8 ± 1.5	12.1 ± 1.4	.28
WBC, /µL	8468.0 ± 2492.7	7864.2 ± 2160.9	.15

Note

*P*‐value comparisons across dx‐code categories are based on the *t* test. Propensity score matching was applied.

Abbreviations: Hb, Hemoglobin; MLR, Monocyte‐lymphocyte ratio; MPV, Mean platelet volume; NLR, Neutrophil‐lymphocyte ratio; PLR, Platelet‐lymphocyte ratio; RDW, Red cell distribution width; WBC, White blood cells.

### 3.3 Association between NLR, PLR, and MLR and disease activity markers in PMR

Neutrophil‐to‐lymphocyte ratio level was correlated with WBC (*r* = .504, *P* < .001), CRP (*r* = .572, *P* < .001), uric acid (*r* = –.292, *P* = .004), albumin (*r* = −.335, *P* = .001), alkaline phosphatase (ALP) (*r* = .433, *P* < .001), aspartate transaminase (AST, *r* = .205, *P* = .043), VAS (*r* = .299, *P* = .003), PLR (*r* = .631, *P* < .001), and MLR (*r* = .508, *P* < .001) in patients with PMR (Table 3). PLR level was correlated with hemoglobin (*r* = −.331, *P* = .001), CRP (*r* = .424, *P* < .001), uric acid (*r* = −.304, *P* = .002), albumin (*r* = −.443, *P* < .001), ALP (*r* = .239, *P* = .018), and VAS (*r* = .225, *P* = .026). MLR level was correlated with WBC (*r* = .453, *P* < .001), CRP (*r* = .421, *P* < .001), and albumin (*r* = −.243, *P* = .016). In comparisons according to manifestations, patients with fever had higher NLR (6.98 ± 3.85 vs 4.05 ± 3.08, *P* = .001) and MLR levels than those without a fever (.61 ± .5 vs .36 ± .22, *P* = .043, Table 4). Patients with headache had higher PLR levels than those without headache (293.2 ± 148.3 vs 214.9 ± 104.6, *P* = .014).

**Table 3:** Correlation between disease activity markers and neutrophil‐lymphocyte ratio (NLR), platelet‐lymphocyte ratio (PLR), and monocyte‐lymphocyte ratio (MLR) in patients with polymyalgia rheumatica

Disease activity markers	Correlation coefficient, *r* (*P*‐value)		
	NLR	PLR	MLR
WBC	.504 (<.001)	−.045 (.659)	.453 (<.001)
Hemoglobin	−.125 (.222)	−.331 (.001)	−.185 (.068)
Platelet	−.017 (.87)	.468 (<.001)	−.197 (.052)
ESR	−.03 (.767)	.210 (.038)	.177 (.082)
CRP	.572 (<.001)	.424 (<.001)	.421 (<.001)
Uric acid	−.292 (.004)	−.304 (.002)	−.113 (.268)
Albumin	−.335 (.001)	−.443 (<.001)	−.243 (.016)
ALP	.433 (<.001)	.239 (.018)	.096 (.345)
Bilirubin	.107 (.292)	−.149 (.143)	.079 (.437)
AST	.205 (.043)	.086 (.398)	.019 (.854)
ALT	.166 (.102)	.02 (.845)	.054 (.596)
Visual analogue scale for pain	.299 (.003)	.225 (.026)	.17 (.094)
Duration of morning stiffness	−.069 (.592)	.007 (.958)	−.011 (.934)
NLR		.631 (<.001)	.508 (<.001)
PLR	.631 (<.001)		.164 (.107)
MLR	.508 (<.001)	.164 (.107)	

Note

These data were assessed using the Pearson correlation test.

Abbreviations: ALP, Alkaline phosphatase; ALT, Alanine aminotransferase; AST, Aspartate transaminase; CRP, C‐reactive protein; ESR, Erythrocyte sedimentation rate; WBC, White blood cells.

**Table 4:** Comparison of neutrophil‐lymphocyte ratio (NLR), platelet‐lymphocyte ratio (PLR), and monocyte‐lymphocyte ratio (MLR) according to manifestations in patients with polymyalgia rheumatica

Manifestations	NLR	*P*‐value	PLR	*P*‐value	MLR	*P*‐value
Fever						
(+), n = 19	6.98 ± 3.85	.001	266.3 ± 108.1	.097	0.61 ± 0.5	.043
(−), n = 79	4.05 ± 3.08		217.4 ± 115.2		0.36 ± 0.22	
Weight loss						
(+), n = 18	5.28 ± 2.83	.365	241.3 ± 101.0	.561	0.57 ± 0.51	.134
(−), n = 80	4.47 ± 3.54		223.7 ± 118.2		0.37 ± 0.24	
Depression						
(+), n = 24	5.31 ± 3.48	.256	249.0 ± 133.1	.280	0.47 ± 0.49	.441
(−), n = 74	4.39 ± 3.40		219.7 ± 108.5		0.39 ± 0.23	
Headache						
(+), n = 15	5.70 ± 3.21	.182	293.2 ± 148.3	.014	0.44 ± 0.16	.649
(−), n = 83	4.42 ± 3.44		214.9 ± 104.6		0.40 ± 0.33	

Note

These data were assessed using the Mann‐Whitney *U* test.

### 3.4 Changes in NLR, PLR, and MLR according to disease activity in PMR

Table 5 shows follow‐up data of the patients with PMR. After the disease activity resolved, the NLR levels had decreased from 4.67 ± 3.44 to 2.95 ± 2.32 (*P* < .001), the MLR levels from 0.41 ± 0.31 to 0.26 ± 0.16 (*P* < .001), and the PLR levels from 229.1 ± 115.1 to 137.5 ± 82.3 (*P* < .001). Changes in NLR were correlated with changes in WBC (*r* = .544, *P* < .001), CRP (*r* = .495 *P* < .001), MLR (*r* = .466, *P* < .001), and PLR (*r* = .669, *P* < .001), and changes in PLR were correlated with changes in CRP (*r* = .396, *P* < .001), platelet (*r* = .323, *P* < .001), and MLR (*r* = .276, *P* = .007, Table 6). Changes in MLR were correlated with changes in WBC (*r* = .395, *P* < .001), ESR (*r* = .216, *P* = .034), and CRP (*r* = .465, *P* < .001).

**Table 5:** Comparison of neutrophil‐lymphocyte ratio (NLR), platelet‐lymphocyte ratio (PLR), and monocyte‐lymphocyte ratio (MLR) in patients with active and inactive polymyalgia rheumatic (PMR)

	Active PMR	Inactive PMR	*P*‐value
NLR	4.67 ± 3.44	2.95 ± 2.32	<.001
PLR	229.1 ± 115.1	137.5 ± 82.3	<.001
MLR	0.41 ± 0.31	0.26 ± 0.16	<.001
ESR, mm/h	74.0 ± 25.8	15.6 ± 7.1	<.001
CRP, mg/dL	7.12 ± 6.61	0.17 ± 0.19	<.001
RDW, %	14.5 ± 1.9	15.6 ± 2.1	<.001
MPV, fL	7.34 ± 0.86	7.55 ± 0.89	.003

Note

These data were assessed using the independent *t* test.

Abbreviations: CRP, C‐reactive protein; ESR, Erythrocyte sedimentation rate; MPV, Mean platelet volume; RDW, Red cell distribution width.

**Table 6:** Correlation between changes in the neutrophil‐lymphocyte ratio (NLR), platelet‐lymphocyte ratio (PLR), and monocyte‐lymphocyte ratio (MLR) and the change in other serologic markers in patients with polymyalgia rheumatica

Disease activity markers	Correlation coefficient, *r* (*P*‐value)		
	Delta NLR	Delta PLR	Delta MLR
Delta WBC	.544 (<.001)	−.009 (.933)	.395 (<.001)
Delta ESR	−.090 (.381)	.110 (.287)	.216 (.034)
Delta CRP	.495 (<.001)	.396 (<.001)	.465 (<.001)
Delta platelet	−.121 (.240)	.323 (.001)	−.056 (.585)
Delta RDW	−.013 (.897)	−.009 (.930)	−.040 (.696)
Delta MPV	.098 (.344)	−.007 (.948)	.111 (.283)
Delta NLR		.669 (<.001)	.466 (<.001)
Delta PLR	.669 (<.001)		.276 (.007)
Delta MLR	.466 (<.001)	.276 (.007)	

Note

These data were assessed based on the Spearman correlation test.

Abbreviations: CRP, C‐reactive protein; ESR, Erythrocyte sedimentation rate; MPV, Mean platelet volume; RDW, Red cell distribution width; WBC, White blood cell.

### 3.5 Clinical characteristics including the levels of NLR, PLR, and MLR according to prognosis in PMR patients

We evaluated differences in NLR, PLR, and MLR levels between the remission group who had no relapse and chronic course group with relapse manifestations. However, the clinical manifestations, including NLR, PLR, and MLR, did not differ between the two groups (data not shown). Patients with a chronic course of PMR had higher counts of swollen joints (2.26 ± 4.97 vs 0.97 ± 2.73, *P* = .03) and more frequent flares (2.07 ± 1.63 vs 0.41 ± 0.6, *P* < .001) compared with those with remission.

## 4 DISCUSSION

This study compared clinical data for 94 patients with PMR with patients with RA, and we showed that NLR, PLR, and MLR were increased in PMR compared with RA, while the difference was not significant when evaluating ESR and CRP levels. NLR was correlated with several laboratory markers, including WBC, CRP, uric acid, albumin, ALP, AST, and VAS in patients with PMR. PLR was correlated with hemoglobin, platelet, ESR, CRP, uric acid, albumin, ALP, and VAS. MLR was correlated with WBC, CRP, and albumin. Patients with fever had higher NLR and MLR compared with patients without fever, while patients with headache had higher PLR compared with patients without headache in patients with PMR. After disease activity resolved, NLR, MLR, and PLR decreased and the changes in NLR and MLR were correlated with changes in leukocytes and CRP. However, NLR, PLR, and MLR did not differ between the remission and chronic course groups in patients with PMR.

Although patients with PMR had fewer tender or swollen joints than patients with RA, pain scores and levels of acute‐phase reactants were higher in patients with PMR. These differences are derived from different characteristics of the two diseases; RA is involved in various peripheral joints, whereas PMR involves a small number of large joints and elevated inflammatory levels are essential for diagnosis. Patients with PMR had higher NLR, PLR, and MLR than patients with RA when they were matched by sex and age, but NLR, PLR, and MLR were not different between the groups when they were matched by CRP and ESR levels. CRP is known to be more specific and sensitive than ESR in assessing PMR disease activity.^3^, ^26^ As high levels of CRP are indicative of an inflammatory response involving infectious or non‐infectious disorders, increased NLR, PLR, and MLR levels also show an activated inflammatory state in patients with PMR. Moreover, changes in CRP were correlated with changes in NLR, PLR, and MLR in follow‐up data of patients with PMR. Combined values of NLR or PLR and CRP have been studied as a more reliable marker to predict prognosis or evaluate the severity of malignant or infectious diseases.^27^, ^28^

Constitutional symptoms including fever, weight loss, and headache occur in 40%‐50% of patients with PMR, and fever is known to be a characteristic symptom of isolated PMR.^3^ Patients with a fever had higher levels of NLR and MLR compared with those without fever. Increased neutrophils and monocytes are believed to contribute to the fever in patients with PMR. The small proportion (15/93) of patients with headache showed increased levels of PLR compared with those without headache. An elevation in platelets may be associated with the headache, and previous studies have observed a relationship between platelets and headache.^29^ Patients with migraine, a primary form of headache, had higher platelet counts and lower platelet membrane fluidity or activity compared with controls.^30^, ^31^ Platelet‐leukocyte interaction releases inflammatory mediators, including interleukins, and platelet serotonin and nitric oxide are known to be secreted and cause headache symptoms.^32^, ^33^

Whether a patient with PMR reaches remission or continues with chronic progression is important in establishing treatment plans or selecting medications. Some patients maintain no or low disease activity, and others relapse with active manifestations of PMR. High levels of initial CRP and ESR are known to be the risk factors indicating recurrent relapse or a chronic course in patients with PMR. However, our results showed no difference in CRP or ESR between the remission and chronic course group. While NLR and MLR were not different, PLR was minimally increased in patients with remission compared with those with a chronic course (*P* = .073). The counts of swollen joints were increased in patients with a chronic course compared with those with remission, which is consistent with previous results showing an association of peripheral arthritis with PMR relapse.

This study had several limitations due to the research method and the study population. There was no biopsy‐proven giant cell arteritis (GCA), and GCA could not be analyzed. While GCA is a major comorbidity in patients with PMR, it is 20 times less common in Asian populations compared with Caucasian patients.^34^, ^35^ This study represents only the characteristics of Asian patients with PMR. In addition, we reviewed the clinical data retrospectively, so the data from some patients were missing. Furthermore, there may have been selection bias since the data were obtained from a single center.

In conclusion, patients with PMR had higher NLR, PLR, and MLR than patients with RA. NLR, PLR, and MLR levels were associated with CRP and albumin in patients with PMR. Fever in patients with PMR was associated with elevated NLR and MLR, and headache was associated with elevated PLR. After disease activity improved, NLR, MLR, and PLR levels decreased. Therefore, NLR and PLR levels at diagnosis are associated with disease activity in patients with PMR.

## CONFLICT OF INTEREST

No potential conflict of interest relevant to this article was reported.
